# Epidemic Assistance by the Epidemic Intelligence Service, Centers for Disease Control and Prevention, 2005–2014

**DOI:** 10.19104/jepm.2016.116

**Published:** 2016-08

**Authors:** Fátima Coronado, Guan M. Chen, Stacey A. Bosch, Danice K. Eaton

**Affiliations:** Division of Scientific Education and Professional Development, Center for Surveillance, Epidemiology, and Laboratory Services, Centers for Disease Control and Prevention, Atlanta, Georgia, USA

**Keywords:** Outbreaks, Epidemiologic Investigations

## Abstract

**Background:**

Epi-Aids, or epidemiologic assistance investigations, are an important mechanism through which Centers for Disease Control and Prevention supports public health organizations. We described the characteristics of Epi-Aids conducted during 2005–2014 and summarized the publication outcome of Epi-Aid related scientific information products.

**Methods:**

We performed a descriptive analysis of all Epi-Aids conducted during January 1, 2005–December 31, 2014; investigations were categorized by health topic and geographic distribution. We highlighted investigations of substantial public health importance, e.g., multistate investigations and investigations of epidemics and pandemics. We identified the Epi-Aid publication outcome by searching PubMed for Epi-Aid related publications, which included Morbidity and Mortality Weekly Reports (MMWRs) and peer-reviewed publications with an Epidemic Intelligence Service Officer (EISO) as a coauthor. We calculated publication timeliness and categorized publications by journal impact factor.

**Results:**

During the study period, 698 EISOs and their collaborators participated in 807 Epi-Aids throughout the United States and globally. Approximately 81 Epi-Aids were conducted annually (range, 62–104); 632 (78.3%) were infectious disease-related; 161 (20.0%) were international, supporting 68 countries. As of June 2015, EISOs, in collaboration with partners, published 131 MMWRs and 280 scientific manuscripts on the basis of the 807 Epi-Aids conducted during the study period; 394 (48.8%) Epi-Aids resulted in publications in 80 peer-reviewed journals.

**Conclusions:**

EISOs play a critical role in conducting Epi-Aids, which require qualified field epidemiologists who can rapidly respond to requests for assistance during public health emergencies. Publications based on Epi-Aids share new knowledge with the scientific community, furthering progress of public health science and practice.

## Introduction

The Epidemic Intelligence Service (EIS) is a 2-year combined training and service fellowship program for health professionals interested in the practice of applied epidemiology [1,2]. The EIS program was established at the Centers for Disease Control and Prevention (CDC) by Alexander Langmuir in 1951 to provide formalized epidemiologic support to health departments and training for the future public health workforce [2,3]. Approximately 80 EIS officers (EISOs) are chosen from > 500 applicants each year and assigned to specific positions at CDC headquarters, state or local health departments, or other organizations (i.e., field-based EISOs) [4]. Field-based EISOs directly support the host jurisdiction with investigations of local health problems and in addition, supplement efforts to strengthen the organization’s public health infrastructure. During their fellowship, EISOs participate in applied, competency-based training through both classroom (~5%) and on-the-job (~95%) experience and are required to complete a set of specific core activities, which include the following: participation in a public health-related field investigation; preparation and submission of a conference abstract, brief scientific report (e.g., for CDC’s Morbidity and Mortality Weekly Report [MMWR]), and manuscript; and presentation to a scientific audience, among others [2,4].

EISOs learn epidemiology and public health practice through service; a fundamental experiential training for EISOs is providing short-term epidemiologic assistance for investigations of serious and urgent public health problems. This assistance is provided in response to formal requests from state, local, federal, and international organizations, and foreign ministries of health (Epi-Aids) [3]. The goal of Epi-Aids is to identify prevention and control measures in response to an urgent outbreak or other public health problem. Epi-Aids are always performed collaboratively with partners, both domestically and internationally, to provide frontline response for investigating disease outbreaks, natural and man-made disasters, and other public health emergencies through use of epidemiologic methods [1,3]. The Epi-Aid team typically consists of at least one EISO not assigned to the inviting jurisdiction, other CDC staff or trainees as needed (e.g., students that participate in the CDC’s Epidemiology Elective), and is supervised by a subject matter expert from CDC headquarters [5]. Since establishment of the EIS program, > 3,500 EISOs have participated in Epi-Aids in the United States and throughout the world [4].

To request an Epi-Aid, public health agencies or organizations formally invite CDC to assist in the investigations [5]. In certain circumstances, Epi-Aids support investigations of health problems affecting more than one state or jurisdiction (multistate Epi-Aid) or affecting the entire country (nationwide Epi-Aid). Epi-Aid responses are documented by the request for an Epi-Aid (i.e., Epi-1), followed by a report of the investigation [3]. The Epi-1 is a form that contains information regarding the subject and nature of the problem, location, persons and organizations involved, and mission objectives. The Epi-Aid report is a written summary produced after each Epi-Aid investigation and provides scientific feedback to the state or locality where the epidemic or health problem occurred; these reports become CDC records. Although the primary objective of Epi-Aids is identification of prevention and control measures to stop the outbreak or public health problem, Epi-Aid reports and the associated knowledge gained often establish the basis for subsequent scientific literature submitted to peer-reviewed journals and MMWR.

Epi-Aids are an important mechanism through which CDC carries out its mission-critical activity of supporting public health departments and other organizations. Although a previous publication summarized Epi-Aids during 1945–2005 [3], publication and report outcomes of these Epi-Aids are not regularly tracked. In this report, we describe the characteristics of Epi-Aid investigations conducted during 2005–2014, highlight key investigations, and summarize the publication outcome of scientific information products developed by EISOs in collaboration with external partners, through documenting Epi-Aid investigations.

## Methods

We performed a descriptive analysis of all Epi-Aid investigations conducted during January 1, 2005–December 31, 2014, by assessing data captured in the EIS Epi-Aid database and Epi-1 request documents. To assess differences in health topics investigated, we categorized the nature of the investigation into infectious disease, chronic disease, environmental health-related, injury-related, and other. The geographic distribution of Epi-Aids was categorized into domestic (investigations conducted in U.S. states, the District of Columbia, and U.S. territories, including tribes and other federal agencies) or international (investigations conducted in other countries or supporting foreign health organizations). We highlighted investigations of substantial public health importance, e.g., multistate investigations and investigations of epidemics and pandemics.

To identify the Epi-Aid publication outcome, we accessed the National Library of Medicine’s database of biomedical literature (PubMed, available at http://www.ncbi.nlm.nih.gov/pubmed), and searched for Epi-Aid related publications on the basis of data from the EIS Epi-Aid database and Epi-1 request documents. The search included MMWRs and peer-reviewed publications with an EISO as a coauthor. When necessary, a Google Scholar® (Google, Incorporated, Mountain View, California) or Scopus® (Elsevier V.B., Amsterdam, Netherlands) search was conducted to identify the number of citations. To assess the dissemination timeliness of these publications, we calculated the number of days from the Epi-Aid initiation date to publication date; if the exact publication date was unavailable, the 15^th^ day of the specified month was used. MMWRs and manuscripts on the basis of Epi-Aids conducted during the study period and published before June 2015 were included in our study; if both the electronic publication and print dates were available, the earlier date was chosen.

Publications were categorized by their respective journal impact factor (IF), obtained from the 2014 Journal Citation Reports® (2013 Journal Impact Factor, Thomson Reuters, Rochester, New York). (JCR) [5]. An impact factor is a rating determined by using the number of times a journal’s articles are cited, providing a measure of the journal’s credibility within the science community. Journal IFs were available only for publications indexed in the JCR for > 2 years. Data were analyzed during June 2015 by using Microsoft® Excel® software (Microsoft Corporation, Redmond, Washington). This project was reviewed by CDC for human subjects protection and was deemed to be nonresearch.

## Results

During January 1, 2005–December 31, 2014, a total of 698 EISOs and their collaborators participated in 807 Epi-Aid investigations throughout the United States and globally. Approximately 81 Epi-Aids were conducted each year (median, 80; range, 62–104) with the highest annual number of Epi-Aids being requested during 2009 and the lowest during 2007 (Table 1). On average, two EISOs were deployed for each Epi-Aid, for a total of 1,522 EISO deployments. The average number of EISOs on the Epi-Aid team increased from one (median, 1; range, 1–4) in 2005 to four (median, 3; range, 1–22) during 2014. Six Epi-Aids involved ≥ 10 EISOs each, which corresponded to substantial public health responses, including the Ebola outbreak response in West Africa during 2014; Middle East respiratory syndrome coronavirus (MERS-CoV) in the United States during 2014; evaluation of pertussis vaccine effectiveness in Vermont during 2014; and influenza A (pH1N1) outbreak in the United States during 2009.

Among the total 807 Epi-Aid investigations, 632 (78.3%) were infectious disease-related (Table 1). The distribution of health topics was similar during each study year, except for 2009 when the percentage of infectious disease-related investigations (85.6%) was higher than other years (Table 1). In 2009, CDC responded to 28 (27%) Epi-Aids in response to the 2009 influenza A (pH1N1) pandemic, including six multistate and nationwide investigations conducted by 72 EISOs. During 2012, CDC responded to a higher proportion of chronic disease-related investigations (7.6%) compared with other years (Table 1). These investigation topics included newborn screening for critical congenital health disease in the states of New Jersey and Georgia [6,7] and investigation of factors associated with disparities in cancer incidence. A higher number of environmental-related investigations (n = 11) were conducted in 2005 compared with other years. The year 2005 is designated the most active Atlantic hurricane season in recorded history and presented seven major hurricanes [8]. CDC responded to six related Epi-Aid requests, including three Epi-Aids in response to Hurricane Katrina supporting emergency preparedness, mortality surveillance [9], carbon monoxide poisoning, and environmental damages. The highest number of injury-related investigations (n = 10) during our study period were conducted during 2008 and addressed topics including peripheral inflammatory neuropathy cases associated with swine slaughter activities [10,11], vascular access hemorrhage among hemodialysis patients [12], and injection safety and control assessment of ambulatory surgical centers [13].

The majority of Epi-Aids in our study were conducted in the United States (80.0%); 87 (10.8%) Epi-Aids were multistate or nationwide investigations; and 37 were conducted in U.S. territories. During 2008, the percentage of domestic investigations (88.4%) was higher than other years. In 2008, seven Epi-Aids were requested and 14 EISOs participated in response to a multistate outbreak of *Salmonella* Saintpaul infections associated with consumption of raw produce, which infected > 1,440 persons in the United States [14–16]. In 2007, an increased number of multistate and nationwide Epi-Aids were conducted (n=10), including a multistate outbreak of *Acanthamoeba* keratitis associated with use of a contact lens solution [17], a multijurisdictional investigation of traveler with extensively drug-resistant tuberculosis, and a multistate outbreak of *Escherichia coli* O157:H7 infection associated with consumption of prepackaged spinach. In 2012, four Epi-Aid investigations were conducted by 12 EISOs in response to a fungal meningitis outbreak among patients who received contaminated steroid injections [18–21]. During 2006, a multistate outbreak of mumps affecting 11 states resulted in three Epi-Aids and deployment of six EISOs [22–24]; also in 2006, two other Epi-Aids were conducted to assist during multistate outbreaks of *Salmonella* infections (i.e., *Salmonella* Newport, *Salmonella* Braenderup, and *Salmonella* Typhimurium) associated with consumption of raw tomatoes eaten in restaurants in the United States [25]. These *Samonella* outbreaks involved 21 states and resulted in the identification of 459 culture-confirmed cases of salmonellosis.

**Figure 1:**
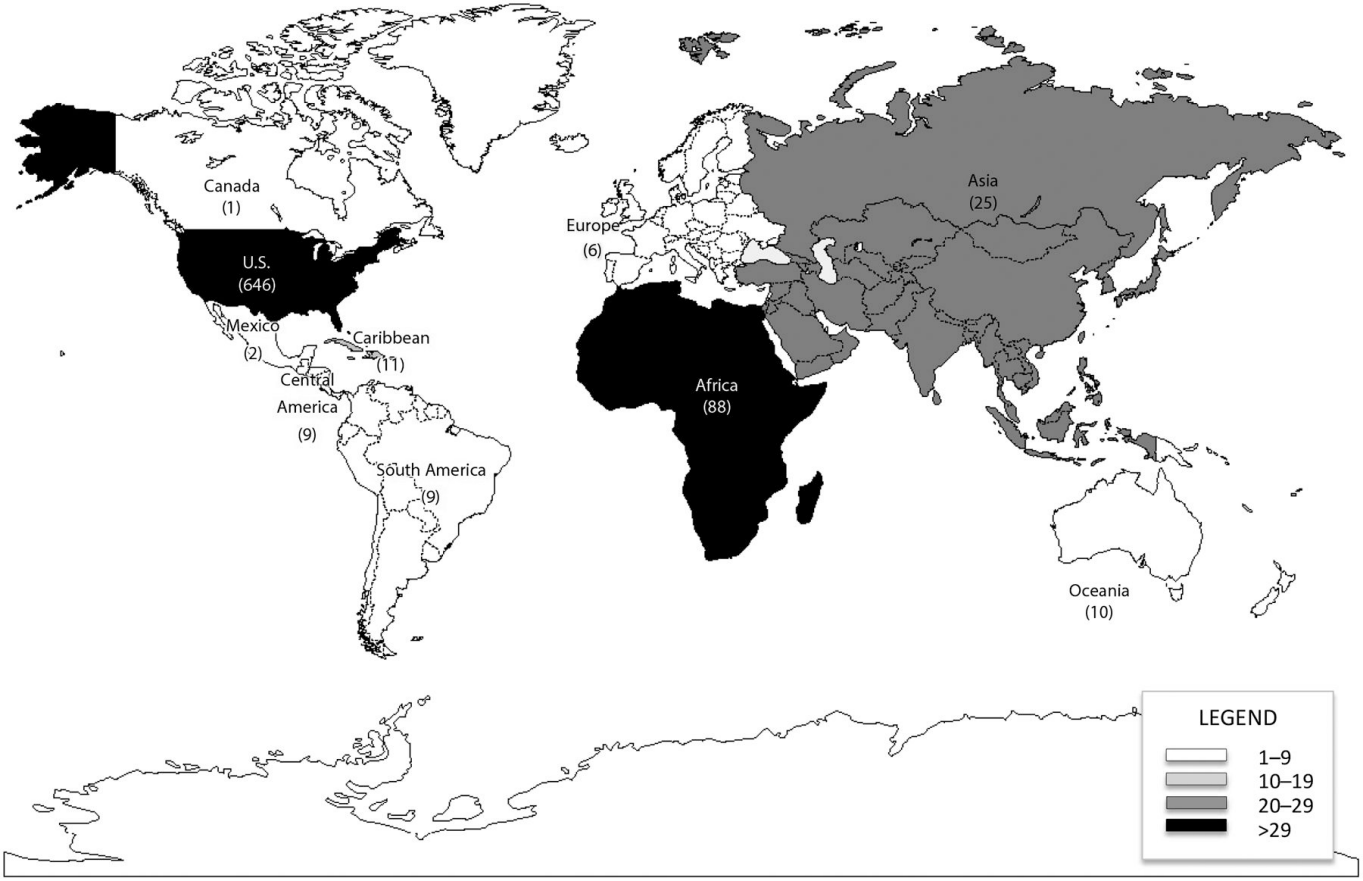
Number of Epi-Aids worldwide, 2005–2014. The U.S. total includes U.S. states, the District of Columbia, and U.S. territories.

A total of 161 (20.0%) Epi-Aids were international and provided support to 68 countries (Figure 1). Epi-Aid requests from international health authorities increased from 10 during 2005 to 17 during 2014, with the majority of requests (n = 88, 55%) from 25 African countries. During 2010, CDC assisted in 97 Epi-Aids, including 25 (25.8%) international investigations, which is approximately twice as many international investigations compared with previous years (Table 1). During the aftermath of the influence A (pH1N1) pandemic, CDC received six Epi-Aid requests from ministries of health to assist in strengthening influenza surveillance systems in Dominican Republic, El Salvador, Nicaragua, Argentina, and South Africa (Johannesburg and Pietermaritzburg). In addition, during January 2010, Haiti experienced a devastating earthquake followed by the world’s largest cholera outbreak [26]. Haiti requested an Epi-Aid for which six EISOs were deployed to investigate the outbreak, support implementation of cholera prevention and control strategies, and evaluate surveillance and response efforts [27]. The highest percentage of international investigations (27.4%) during our study period was conducted in 2014. Three Epi-Aids were requested after the first local transmission of emerging chikungunya virus in the Americas was identified on Caribbean islands during late 2013 [28,29]. In 2014, the largest Ebola epidemic in history was reported and affected multiple West African countries; > 27,000 cases and > 11,000 deaths were reported from Guinea, Sierra Leone, and Liberia [30]. In the United States, two imported cases that included one death, and two locally acquired cases among healthcare workers were reported. In response to the Ebola outbreaks, eight Epi-Aids were conducted by 46 EISOs and their colleagues in four countries in West Africa and two U.S. states [31–34].

### Publications on the Basis of Epi-Aids

As of June 2015, CDC EISOs, in collaboration with external public health partners, have published 411 scientific information products, including 131 MMWRs and 280 scientific manuscripts on the basis of Epi-Aid investigations conducted during January 1, 2005–December 31, 2014 (Table 2). Among 807 Epi-Aids, 394 (48.8%) investigations resulted in publications; 347 (43%) Epi-Aids resulted in one publication, 42 (5.2%) resulted in two publications, four (0.5%) resulted in three publications, and a 2006 investigation of respirator use in water-damaged buildings in Louisiana resulted in four (0.1%) publications (data not displayed). On the basis of Epi-Aids each year, EISOs published an average of 13 MMWRs (median, 13; range, 5–25) and 28 (median, 30; range, 2–46) scientific manuscripts. The majority of publications were infectious disease-related (81%), with a higher percentage of infectious disease-related manuscripts (83.2%) than MMWRs (76.3%). The median time from Epi-Aid initiation date to publication was 196 days (mean time, 262 days; range, 11–1,398 days) for *MMWRs* and 828 days (mean time, 926 days; range, 82–2,858 days) for manuscripts. A total of 110 (39%) manuscripts were published within two years of Epi-Aid initiation, and 110 (84%) MMWRs were published within one year of Epi-Aid initiation.

EISOs collaborated with public health authorities in the inviting jurisdictions to publish findings from Epi-Aid investigations in a total of 80 peer-reviewed medical, public health, and state medical journals. The majority of manuscripts (55%) were published in a journal with an IF of 1–5; a total of 31 (11%) manuscripts were published in journals with IFs from 1 to < 2; and 49 (18%) manuscripts were published in journals with IFs from ≥ 2 to 3. Nineteen (7%) manuscripts were published in a journal with an IF of > 20. Manuscripts on the basis of Epi-Aids were most commonly published in Clinical Infectious Diseases (11.4%), Emerging Infectious Diseases (8.2%), and Infection Control and Hospital Epidemiology (7.5%).

Among the Epi-Aid investigations published, EISOs served as first author on 240 (86%) manuscripts, and as coauthors on 40 (14%) manuscripts; the manuscripts published during the study period were cited 6,536 times, approximately 23 times per manuscript (median, 11; range, 0–450), with 252 (90%) manuscripts cited at least once. The manuscript most cited was about an investigation of patterns of abuse among unintentional pharmaceutical overdose fatalities and was cited 450 times since its publication in 2008 [35]. The majority of manuscripts published during 2015 (12 of 15; 82%) have not been cited yet. The 131 MMWRs published during the study period were cited 2,598 times, approximately 20 times per MMWR (median, 9; range, 0–125). The MMWR most cited resulted from a 2009 influenza A (H1N1) pandemic investigation, and was cited 125 times [36].

## Discussion

EISOs are a vital element at the frontline of public health, conducting epidemiologic investigations, research, and surveillance-related activities, both domestically and internationally [1]. EISOs also play a critical role in conducting Epi-Aid investigations, which require a pool of field-based epidemiologists who can respond rapidly during a public health emergency. Since establishment of the EIS program, EISOs have provided valuable service to public health agencies through their participation in > 5,000 Epi-Aid investigations. During the past 10 years, EISOs have led > 800 Epi-Aids, not including the service provided by EIS field-based officers and other CDC staff assigned to states, local health departments, and international settings outside the formal Epi-Aid mechanism; this includes 600–800 field investigations conducted annually, which is > 6 times the number of Epi-Aids conducted each year [4].

Through the Epi-Aid mechanism, EISOs provide technical expertise for rapid response to outbreaks and other public health emergencies; streamline access to CDC subject matter expertise, laboratory, and other resources; and strengthen epidemiologic capacity in the jurisdiction requesting assistance. Epi-Aids also offer instrumental learning opportunities to EISOs regarding application of epidemiologic methods and public health interventions. In addition, during public health emergencies (e.g., Hurricane Katrina, 2009 influenza A (pH1N1) pandemic, and Ebola epidemic), CDC’s Emergency Operations Center (EOC), the command center for monitoring and coordinating response activities to public health threats in the United States and internationally, is activated. The EOC serves as a centralized component that provides EISOs and CDC staff the resources needed to effectively carry out public health response. For example, during the EOC activation in response to the 2009 influenza A (pH1N1) pandemic, > 3,300 CDC staff, including EISOs, supported this international response [37].

The majority of Epi-Aids in our study were conducted in response to infectious disease-related threats. Infectious disease remains a primary concern to public health agencies with health departments spending approximately $58 million/year preventing infectious diseases [38]. To serve public health needs, CDC has a capable and qualified workforce that can offer expertise during these investigations, with approximately 30% of this workforce located in operational units with subject matter expertise regarding infectious diseases [39]. Nevertheless, CDC has broadened its capability by adding programs in reproductive health, chronic diseases, injuries, environmental health exposure, and other noninfectious conditions [3]. Epi-Aid investigations have evolved similarly and become increasingly focused on the prevention and control of noninfectious diseases. Before 1960, only 8% of Epi-Aids were noninfectious disease-related; this percentage increased to 22% during 2005–2014.

During the past 60 years, the geographic scope of Epi-Aid investigations has broadened and an increase in support to international health authorities has been observed. During 2005–2014, the number of international investigations increased to approximately 20%, compared with only 11% during 1946–2005 [3]. This increase might be attributed to increased visibility of the EIS program and the recognition of Epi-Aids worldwide. Because persons and pathogens travel across borders more freely, we anticipate an increased number of requests for CDC assistance and Epi-Aids with focus on global health concerns; further, we anticipate an increase in the number of investigations involving multiple countries, as demonstrated by the Ebola outbreak in West Africa.

Although Epi-Aids are both effective and beneficial, these investigations do not represent the full response of CDC or the EIS program. CDC staff and EISOs are often deployed outside the Epi-Aid mechanism to assist in outbreak investigations. For example, as of November 2015, approximately 2,000 CDC staff and EISOs participated in > 2,500 field deployments to assist with Ebola response efforts that included surveillance, contact tracing, data management, laboratory testing, and health education across the United States and Africa (CDC administrative data). In addition, hundreds of CDC staff were stationed at the EOC to provide logistics, staffing, communication, analytics, management, and other Ebola response support functions.

Besides demonstrating expertise in epidemiologic investigations, EISOs are expected to attain prescribed competencies in scientific communications. After completing an Epi-Aid investigation, EISOs submit a report to the pertinent public health authority that clearly presents findings and recommendations to guide decisions, policies, and public health action. As part of their training, EISOs are encouraged to collaborate with field partners to publish Epi-Aid investigation results. During our study period, EISOs published > 400 scientific information products on the basis of Epi-Aids, and approximately half of the Epi-Aids resulted in ≥ 1 publication. Although the primary objective of Epi-Aids is to prevent and control public health problems, the resultant publications share new knowledge, lessons learned, and recommendations with the scientific community, furthering progress of public health science, practice, and policy.

Our study has certain limitations. First, because of the high level of scrutiny and extensive peer-review process required before publication, the analyses focused on MMWRs and manuscripts published in peer-reviewed journals. Our study did not include abstracts, which although cleared by CDC, are not peer-reviewed and are difficult to compare across different conferences. Second, our study might underestimate the number of publications, particularly for Epi-Aids conducted during 2014. Given that it can take months after journal acceptance for MMWRs and manuscripts to be published, certain Epi-Aids might not have been captured in the analyses or might have been missed if not clearly associated with an Epi-Aid or EISO coauthor. Third, we lacked information regarding the date of Epi-Aid completion, which would be a more accurate way to measure the time necessary for an EISO and collaborators to publish their work on the basis of Epi-Aids. Finally, our study included publications on the basis of Epi-Aids with EISOs as coauthors, which might underestimate the total number of publications available on the basis of Epi-Aids.

The EIS program has served the United States and the world for > 60 years and will continue to play a critical role on behalf of CDC in supporting our public health partners in investigating public health concerns. Although Epi-Aids are only a part of work performed by EISOs, they represent a substantial contribution of epidemiologic assistance to the United States and international community. Collectively, Epi-Aids provide knowledge transfer and also offer EISOs an avenue for broader experience and training in epidemiologic methods, communications, and leadership. Because new public health problems emerge and the practice of applied epidemiology expands to new areas [1], EISO participation and contribution in epidemiologic field investigations are valuable in helping protect the public’s health.

## Figures and Tables

**Table 1:** Characteristics of Epi-Aid investigations conducted by EISOs, by year — 2005–2014.

	2005	2006	2007	2008	2009	2010	2011	2012	2013	2014	Total
**Health topic, no. (%)**											
Infectious disease	59 (75.6)	59 (79.7)	48 (77.4)	66 (76.7)	89 (85.6)	75 (77.3)	65 (77.4)	58 (73.4)	65 (80.2)	49 (79.0)	**632 (78.3)**
Chronic disease	2 (2.6)	3 (4.1)	2 (3.2)	3 (3.5)	4(3.8)	2 (2.1)	4 (4.8)	6 (7.6)	6 (7.4)	3 (4.8)	**35 (4.3)**
Environmental-related	11 (14.1)	8 (10.8)	6 (9.7)	5 (5.8)	7 (6.7)	9 (9.3)	6 (7.1)	3 (3.8)	6 (7.4)	4 (6.5)	**65 (8.1)**
Injury-related	1 (1.3)	3 (4.1)	5 (8.1)	10 (11.6)	2 (1.9)	3 (3.1)	3 (3.6)	5 (6.3)	1 (1.2)	5 (8.1)	**38 (4.7)**
Other subject matter*	5 (6.4)	1 (1.4)	1 (1.6)	2 (2.3)	2 (1.9)	8 (8.2)	6 (7.1)	7 (8.9)	3 (3.7)	1 (1.6)	**36 (4.5)**
**Geographic Scope, no. (%)**											
Domestic											
Multistate or nationwide	6 (7.7)	6 (8.1)	10 (16.1)	9 (10.5)	15 (14.4)	7 (7.2)	12 (14.3)	9 (11.4)	8 (9.9)	5 (8.1)	**87 (10.8)**
Single state or location	62 (79.5)	55 (74.3)	41 (66.1)	67 (77.9)	76 (73.1)	65 (67.0)	51 (60.7)	49 (62.0)	53 (65.4)	40 (64.5)	**559 (69.3)**
International	10 (12.8)	13 (17.6)	11 (17.7)	10 (11.6)	13 (12.5)	25 (25.8)	21 (25.0)	21 (26.6)	20 (24.7)	17 (27.4)	**161 (20.0)**
**Total**	**78 (9.7)**	**74 (9.2)**	**62 (7.7)**	**86 (10.7)**	**104 (12.9)**	**97 (12.0)**	**84 (10.4)**	**79 (9.8)**	**81 (10.0)**	**62 (7.7)**	**807 (100)**

EIS = Epidemic Intelligence Service; Epi-Aid = epidemiologic assistance investigation. Note: Percentages might not total 100 because of rounding.

* Other subject matter includes emergency preparedness, public health workforce education and training, unexplained illness, and multiple topics.

**Table 2:** Peer -reviewed scientific products on the basis of Epi-Aids conducted during 2005–2014, by product type — 2005–2014.

	MMWR (N = 131)	Manuscripts (N = 280)
**Health topic, no. (%)** Infectious disease	100 (76.3)	233 (83.2)
Chronic disease	12 (9.2)	5 (1.8)
Environmental-related	5 (3.8)	25 (8.9)
Injury-related	7 (5.3)	11 (3.9)
Other subject matter*	7 (5.3)	6 (2.1)
**Time from Epi-Aid initiation to publication, days**		
Mean	262	926
Median (Range)	196 (11–1,398)	828 (82–2,858)

EIS = Epidemic Intelligence Service; Epi-Aid = epidemiologic assistance investigation; *MMWR* = *Morbidity and Mortality Weekly Report*.

* Other subject matter includes emergency preparedness, public health workforce education and training, unexplained illness, and multiple topics.
